# Autonomic Nervous System and Recall Modeling in Audiovisual Emotion-Mediated Advertising Using Partial Least Squares-Path Modeling

**DOI:** 10.3389/fpsyg.2020.576771

**Published:** 2020-10-30

**Authors:** Óscar Barquero-Pérez, Miguel Angel Cámara-Vázquez, Alba Vadillo-Valderrama, Rebeca Goya-Esteban

**Affiliations:** Signal Theory and Communications Department, University Rey Juan Carlos, Madrid, Spain

**Keywords:** neuromarketing, autonomic nervous system, emotion, recall, heart rate variability, electrodermal activity, partial least squares-path modeling

## Abstract

Interest in improving advertisement impact on potential consumers has increased recently. One well-known strategy is to use emotion-based advertisement. In this approach, an emotional link with consumers is created, aiming to enhance the memorization process. In recent years, Neuromarketing techniques have allowed us to obtain more objective information on this process. However, the role of the autonomic nervous system (ANS) in the memorization process using emotional advertisement still needs further research. In this work, we propose the use of two physiological signals, namely, an electrocardiogram (heart rate variability, HRV) and electrodermal activity (EDA), to obtain indices assessing the ANS. We measured these signals in 43 subjects during the observation of six different spots, each conveying a different emotion (rational, disgust, anger, surprise, and sadness). After observing the spots, subjects were asked to answer a questionnaire to measure the spontaneous and induced recall. We propose the use of a statistical data-driven model based on Partial Least Squares-Path Modeling (PSL-PM), which allows us to incorporate contextual knowledge by defining a relational graph of unobservable variables (latent variables, LV), which are, in turn, estimated by measured variables (indices of the ANS). We defined four LVs, namely, *sympathetic, vagal, ANS*, and *recall*. *Sympathetic* and *vagal* are connected to the *ANS*, the latter being a measure of recall, estimated from a questionnaire. The model is then fitted to the data. Results showed that vagal activity (described by HRV indices) is the most critical factor to describe ANS activity; they are inversely related except for the spot, which is mainly rational. The model captured a moderate-to-high variability of ANS behavior, ranging from 38% up to 64% of the explained variance of the ANS. However, it can explain at most 11% of the recall score of the subjects. The proposed approach allows for the easy inclusion of more physiological measurements and provides an easy-to-interpret model of the ANS response to emotional advertisement.

## 1. Introduction

Interest in improving advertisement impact on potential consumers has increased recently. In this context, Neuromarketing techniques have allowed us to obtain more objective information on how the consumer unconsciously processes the information (Norton et al., 2007; Babiloni, 2015). In recent years, advertisements have tended to use emotion-based approaches to create an emotional link with consumers; the hypothesis is that this emotional engagement will enhance the memorization process and, eventually, the recall (Roberts, 2004; Vecchiato et al., 2010, 2014; Baraybar-Fernández et al., 2017).

Neuromarketing is the application of neurophysiological tools to assess and objectively understand human behavior concerning marketing product development (Lee et al., 2007; Babiloni, 2015). Nowadays, the main research techniques are the following: (1) neuroimaging technologies to assess brain activity, such as functional magnetic resonances (fMRI), electroencephalogram (EEG), and magnetoencephalogram (MNG), for market research (Ioannides et al., 2000; Vecchiato et al., 2010, 2014); and (2) autonomic nervous system (ANS) analysis tools, such as electrodermal analysis (EDA) and heart rate variability (HRV), to study emotion, cognition, and attention (Critchley, 2002; Cartocci et al., 2017). The latest, EDA and HRV, are physiological signals that can be registered with cheap and easy-to-use equipment.

The neuromarketing approach tries to assess, as objectively as possible, the emotional impact on how the memory is formed during the observation of advertisements. It has been shown that this emotional reaction and perception of the stimuli is mainly unconscious. In this context, HRV and EDA assess the emotional status, as indexes reflecting the ANS activity. Several studies used the HRV and EDA to create an emotional index (EI) based on the theoretical works about emotions, particularly regarding the valence and arousal plane of emotions (Mauss and Robinson, 2009; Babiloni, 2015; Cartocci et al., 2017). Some approaches have combined the sympathetic activity estimated from EDA and vagal activity estimated from HRV to propose a reliable index of the sympathovagal balance in cold stress test studies (Ghiasi et al., 2018, 2020). However, there is still a need for an improved understanding of the relationship between the sympathetic and vagal branches of the ANS and the recall induced by different emotions during observation of advertisements.

Different studies have demonstrated that positive or negative emotion stimuli are an important element when establishing memory traces (Kato, 2009; Baraybar-Fernández et al., 2017; Cartocci et al., 2017). Neuromarketing techniques allow us to objectively measure the reaction of consumers to emotion stimulus in advertisements, and it does so namely by assessing cerebral activity variations (Davidson and Irwin, 1999; Vecchiato et al., 2014; Babiloni, 2015) and ANS status modifications (Babiloni, 2015; Cartocci et al., 2017). The ANS plays an essential role in the emotional status of the subjects viewing advertisements. Different studies suggested an increase in the parasympathetic activity for positive emotions, whereas negative emotions (anger, fear, and sadness) result in parasympathetic withdrawal and sympathetic activation (McCraty et al., 1995; Kop et al., 2011).

In this work, we propose a modeling of the activity of the ANS as a result of modulation by sympathetic and vagal branches. Moreover, we model the impact of the ANS on recall. These are unobservable variables (Latent Variables, LV) that cannot be measured directly. We propose the use of several manifest variables (MV), i.e., directly measured indices, that are related to the LV. In this way, LVs represent abstract concepts that are combinations of the observable variables, i.e., MVs. Since we want to estimate ANS influence on recall, we propose the use of HRV- and EDA-based indices to assess the ANS activity. We used partial least squares path modeling approach (PLS-PM), which is an alternative method to covariance-based estimation for structural equation models (SEM) (Wold, 1975; Tenenhaus et al., 2005).

PLS-PM does not rely on strong assumptions for probability distributions of the variables and error. It is a data-driven approach in which the aim is to provide a model to explain the relationship between LVs and MVs. It can be viewed as an explanatory approach more so than a confirmatory one (Esposito et al., 2010; Sánchez, 2013; Serrano et al., 2014). Both properties, the lack of assumptions, and the explanatory approach are well-suited to address the problem we are facing in this study. We propose an assessment of the ANS by HRV indices, which are known to characterize both sympathetic and vagal branches, as well as by EDA indices, which mainly characterize sympathetic tone (Malik et al., 1996; Boucsein, 2012; Greco et al., 2016). These indices will represent the MVs in the PLS-PM. These physiological variables are measured in subjects during observation of advertisements conveying different emotional stimuli. Once finished the observation, we measured recall using a questionnaire, which evaluates the spontaneous and induced recall. Finally, the PLS-PM was complemented with a recall score for each spot.

The structure of the manuscript is as follows. In section 2, the experimental setup and signal acquisition are described. In section 3, the ANS evaluation using HRV and EDA-based indices is stated. In section 4, the statistical modeling of the ANS and the recall using PLS-PM are described. In section 5, the results of the proposed model are presented. Finally, the discussion and our conclusions are presented in section 6.

## 2. Experimental Setup

The subjects in the experiment were asked to look at a computer screen at first with neutral content (black screen). The physiological recording equipment was correctly placed, and the subject was left for 1 min in this situation to stabilize and serve as a baseline. After that, the subject observed six different advertisements in a row. Once the visualization ended, the subjects were asked to answer a questionnaire that measured both spontaneous and suggested recall.

Six advertising spots of 1 min in length each were used in this experiment. Four of them expressed predominantly one emotion, namely, surprise, sadness, disgust, and anger, and two included formative content (rational messages). The advertisements were selected by a group of eight experts (four academics and four publicists), and the order of the spots was determined randomly to minimize the primacy (first) effect and recency (last) effect. The place of the spots can generate higher recall in the study population (Glanzer and Cunitz, 1966). The final order of the messages was the following: (1) *rational*, traffic speed in cities; (2) *disgust*, cocaine use; (3) *anger*, child abuse; (4) *surprise*, alcohol and driving; (5) *sadness*, speed and distractions on the road; and (6) *rational*, addiction treatments. The videos are part of the Supplementary Material.

The experiment was conducted with a total of 66 participants (30 males and 36 females) aged between 18 and 41 (22.5 ± 4.45 years old). Participants were healthy students of different undergraduate or post-graduate degrees or staff of the Rey Juan Carlos University. All subjects gave their informed consent and were declared to be healthy and without heart conditions. The experiment had the approval of the University ethic committee. ECG and EDA signals were continuously recorded during the experiment. Signals were recorded using BITalino system (Plux Wireless Biosignals S.A. Portugal), with a sampling rate of 1 kHz. The ECG was registered using a derivation from three electrodes, positive and negative on the wrists, and the reference electrode on the forearm of the subject. EDA signal was recorded placing a pair of electrodes on the left palm. After this procedure we manually reviewed the data and discarded subjects with poor signal quality (a lot of artifacts, loose electrodes, interruptions in the recording). The final data set ended up with 43 subjects (25 females).

This questionnaire measures the spontaneous and suggests recall. The questionnaire has three sections:

- Demographic variables: age, gender, education, and nationality.
- Spontaneous recall: participants are asked about their direct remembrance of the main message conveyed in the advertisements as a free text. They are also asked what emotions they felt, in degrees akin to a Lickert scale, and in relation to which advertisements as a free text.
- Suggested recall: different situations are presented, and participants are asked if those situations happened in the spots (yes/no questions). If the answer is *yes*, participants are asked to select the messages they remember from that particular situation. The list of messages includes those that appeared in the spots, among others, as a multiple-choice question.

The complete questionnaire is available in the Supplementary Material in Spanish.

We quantified recall by assigning to each question an individual score, which was maxed out if the subject recalled correctly (emotions and messages conveyed in the advertisements). The final score was the addition of individual scores. The recall-score ranged from 0 (no recall at all) to 15 points (perfect recall).

## 3. ANS Characterization Using HRV and EDA

In this section, the processing of the ECG and EDA signals to obtain ANS markers is described. The aim is to describe the indices we finally used as MV in the final PLS-PM model we propose. The interested reader can find a more detailed description in Malik et al. (1996) and Greco et al. (2016).

### 3.1. ECG-HRV Analysis

The heart behavior is not constant; instead, there exists a variation in the time intervals between consecutive heartbeats. The normal heart rhythm is controlled by the cardiac sinoatrial (SA) node, modulated by innervation from both the sympathetic and the vagal branches of the ANS. The SA node is the final mechanism responsible, through its repetitive nervous impulses, for generating heartbeats.

Both the sympathetic and parasympathetic responses have antagonist roles, i.e., the sympathetic activity increases the heart rate, whereas the vagal activity slows down the heart rate. In rest conditions, there is a dynamic balance state between these systems, which is responsible for the variability in the intervals between consecutive heartbeats. At the same time, the ANS is influenced by many other systems (respiratory system, vasomotor system, central nervous system, or renin-angiotensin system), which also indirectly contribute to modulate the heart rate through it (Malik et al., 1996).

HRV is the variation in the intervals between consecutive heartbeats, and it allows for non-invasive investigation of the ANS state. The most straightforward time signal representation of the HRV is the tachogram, representing the time between consecutive beats (RR-intervals or NN-intervals) vs. the interval number. The NN-intervals are the time distance between R-waves of consecutive beats, excluding the ectopic beats (those that do not have their origin at the SA node).

Several methods have been proposed in the literature to assess HRV. Time-domain and spectral-domain methods are the most widely used indices due to their simplicity and interpretability. A sub-class of time-domain methods, known as statistical methods, involves calculating the standard deviation of either the RR-intervals series or its first difference (Malik et al., 1996; Mietus et al., 2002). Table 1 summarizes the statistical indices computed in this work. Additionally, we use the mean of the NN intervals, AVNN.

**Table 1:** Time and frequency domain indices of HRV.

**Index**	**Units**	**Description**
**TIME-DOMAIN**		
SDNN	ms	Standard deviation of NN intervals.
RMSSD	ms	The Square root of the mean of the sum of the squares of differences between adjacent NN intervals.
pNN50		Number of pairs of adjacent NN intervals differing by more than 50 ms in the entire recording divided by the total number of NN intervals.
**FREQUENCY-DOMAIN**		
LF	*ms*^2^	Power in low frequency range.
		*LF*/(Total power−*VLF*)*100.
HF	*ms*^2^	Power in High frequency range.
		*HF*/(Total power−*VLF*)*100.
LF/HR	adim.	Ratio *LF*[*ms*^2^]/*HF*[*ms*^2^].

Spectral methods indicate how the power of the signal is distributed as a function of the frequency *f*. HRV found in healthy subjects during rest is influenced by respiratory activity and the feedback mechanism of the systems for regulation of temperature and blood pressure. The different systems oscillate spontaneously at rest with characteristic frequencies at different intervals. By quantifying the power of the spectral components, information related to autonomic cardiac function may be pointed out (Cerutti et al., 1995; Persson, 1997; Sörnmo and Laguna, 2005).

Four spectral bands are mainly distinguished in the power spectral density of the NN series, namely, the high frequency (HF) band with *f* ∈ (0.15, 0.4) Hz, low frequency (LF) band with *f* ∈ (0.04, 0.15) Hz, very low frequency (VLF) band with *f* ∈ (0.003, 0.04) Hz, and ultra low frequency (ULF) band with *f* < 0.003 Hz (Akselrod et al., 1991). Frequency-domain indices are calculated based on these spectral bands (Malik et al., 1996). Due to the length limitations of the signals, in this work, we focus on the frequency-domain indices listed in Table 1.

HF power is generally taken as an index of cardiac parasympathetic tone, although some studies have shown that it is, to some degree, also affected by the sympathetic tone and may not be a pure parasympathetic index. The LF index is more controversial. Many studies have pointed out that LF power reflects sympathetic and vagal activity and that the LF/HF ratio reflects sympathovagal balance. In short-term HRV recordings under experimental conditions affecting autonomic response, the relative LF power contribution is often considered a marker of sympathetic activity. However, some studies using pharmacological, physiological, or psychological manipulations affecting the sympathetic activity and HRV have questioned this association. One crucial fact is that vagal blockade strongly reduces LF power, whereas sympathetic blockade has no significant effect. A review of these studies and the corresponding references can be found in Iwase et al. (2017).

### 3.2. EDA–Analysis

The conductance of the skin is regulated by the sweat gland activity. This modulation is the cause of the EDA. In this context, EDA has been viewed as a sensitive and straightforward way to assess the sympathetic arousal associate with emotion (Vecchiato et al., 2014; Posada-Quintero and Chon, 2020). Since sympathetic function directly modulates the sudomotor activity, EDA is indicated to evaluate sympathetic autonomic function. Indeed, several studies have used the EDA signal, along with HRV, to build an emotional index (EI) in the context of Neuromarketing techniques (Cartocci et al., 2017). The EI represents the valence and arousal response to external stimuli, which is mediated by the ANS.

The EDA signal has two main components: the skin conductance level (SCL) and the skin conductance response (SCR). SLC represents the overall conductance of the tonic component of EDA. It is related to slow shifts of the EDA signal (Greco et al., 2016; Posada-Quintero and Chon, 2020). To obtain the tonic component (SCL), a low-pass filter with cut-frequency 0.1 Hz was applied (Vecchiato et al., 2014). We quantified the contribution of the SCL as the mean value during the observation of the advertisement. The other component of the EDA signal, SCR, is the response to external stimuli. To automatically detect the SCR, we used the algorithm proposed in Kim et al. (2004). In this algorithm, the EDA signal is first downsampled to 20 samples per second. The signal is then differentiated to emphasize high-frequency content, and it is finally convolved with a 20-point Bartlett window. This processing produces a spiky signal in which the spikes correspond to SCRs, and simplify the detection procedure. To account for this part, we proposed the use of the number of SCRs (*ns*_*scr*) as an index of the phasic component of the EDA signal for each advertisement.

## 4. ANS and Recall Modeling Using PLS-PM

PLS-PM is an iterative algorithm that estimates the relationship between MVs and LVs by the weights of multiple and simple regressions. PLS-PM allows to also obtain linear relationship between LVs (Wold, 1975; Tenenhaus et al., 2005). A full path model is comprised of two submodels: (1) the measurement model, which establishes the relationship between each LV and its own MVs; and (2) the structure model, which considers the relationship between LVs (Tenenhaus et al., 2005; Sánchez, 2013).

- *LV–Sympathetic*: We associated several indices that, in the scientific literature, are related to the sympathetic activity, namely, from HRV analysis, *lf*, *sdnn*, and, from EDA analysis *ns*_*scr*, *mean*_*scl* (Posada-Quintero et al., 2016; Ghiasi et al., 2020; Posada-Quintero and Chon, 2020). Indices from EDA are clearly related to the sympathetic activity, while the HRV indices raise more controversy about its association to sympathetic and vagal branches (Malik and Camm, 1993; Coumel et al., 1994; Sacha, 2014; Medeiros et al., 2018). However, PLS-PM allows us to measure the adequacy of belonging to each LV, and this allows for the convenience of changing membership to another LV.
- *LV–Vagal*: The MVs associated with this LV are, from HRV analysis, *hf*, *pnn*50, *rmssd*. The evidence from scientific literature supports the relationship between these indices and the Vagal activity (Malik and Camm, 1993; Malik et al., 1996; Medeiros et al., 2018).
- *LV–ANS*: The MVs associated with this LV are, from HRV analysis, the classical indices that assess the sympatho-vagal balance: *lf*_*hf*, *avnn* (Akselrod et al., 1991; Malik and Camm, 1993; Malik et al., 1996).
- *LV–Recall*: This LV represents the recall of the subjects, measured by the recall-score described in section 2.

**Figure 1:**
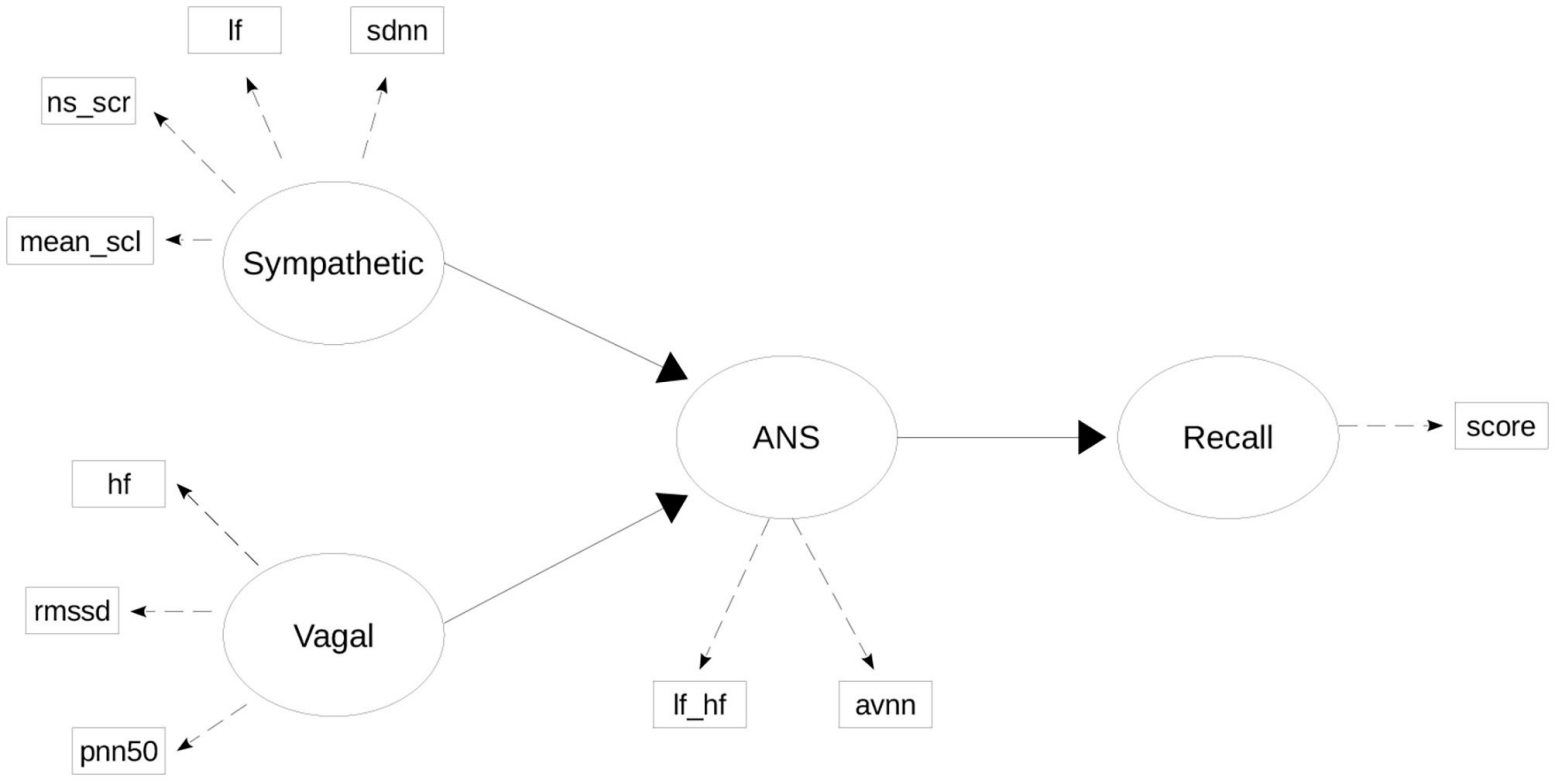
PLS-PM model of the ANS activity and Recall. Dashed lines represent the links in the measurement model, and solid lines represent links in structure model.

Regarding the structural model, it was assumed that the *ANS* directly depends on the *Sympathetic* and *Vagal* LVs, while the *recall* construct depends directly on *ANS*. The complete scheme of the structural and the measurement model can be seen in Figure 1.

The model was designed such that MVs are considered to be caused by the latent variables, i.e., reflective indicators. This assumption imposes a restriction since all the MVs are measuring the same LVs. All MVs therefore have to be highly correlated (Sánchez, 2013). Consequently, some of the MVs had to change their sign to follow along with the remaining MVs. The structure model is statistically represented by two linear regression models: (1) ANS as a function of Sympathetic and Vagal activity and (2) recall as a function of the ANS. The path coefficients (β) were obtained as classical weights in linear regression, i.e., using a least-square approach (Esposito et al., 2010). The overall fit of the final model was assessed by the goodness-of-fit (Tenenhaus et al., 2004). Gender differences are studied using a permutation resampling procedure to determine whether path coefficients are statistically different (Sánchez, 2013).

## 5. Results

In this section, we summarize the most relevant results. Firstly, before the analysis of the PLS-PM models, we present the analysis of the recall scores for the different emotions represented in the spots. Next, we present the results of the measurement model, which allows us to identify the adequacy of the MVs as valid indicators of the LV. Finally, we present the results for the structure model and compare the different results for different emotions represented in the spots.

### 5.1. Recall Scores

**Figure 2:**
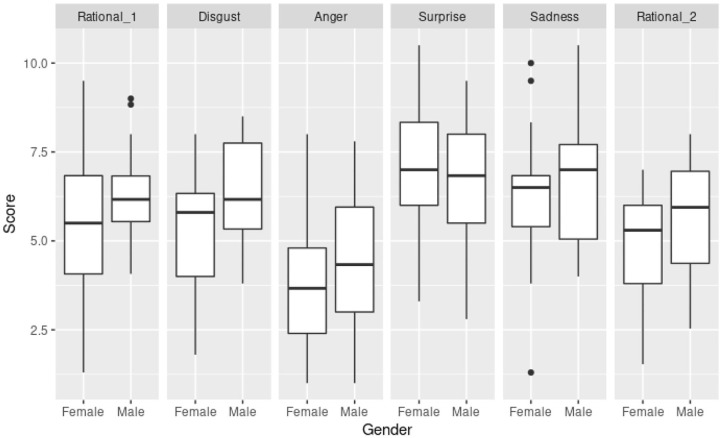
Recall-score distribution grouped by gender and for the different emotions conveyed in the advertisements. Emotions are represented in the same order as the subject watched the spots.

Figure 2 shows the distribution of the recall-score grouped by gender. The final score was composed as points obtained when the subject provided a correct answer in the questionnaire. The recall-score measured both spontaneous and induced recall. In this context, the higher the score, the higher the recall. Most emotions generated a similar recall-score; the only exception was the spot with *Anger* emotion, showing less significant recall-score. The mean value recall-score for all spots but *Anger* was 6.0, while the recall-score for *Anger* spot was 4.0. The difference by gender was non-significant, with slightly higher recall-score values for males, in general.

### 5.2. Structure Model: ANS and Recall PLS-PM Model

**Figure 3:**
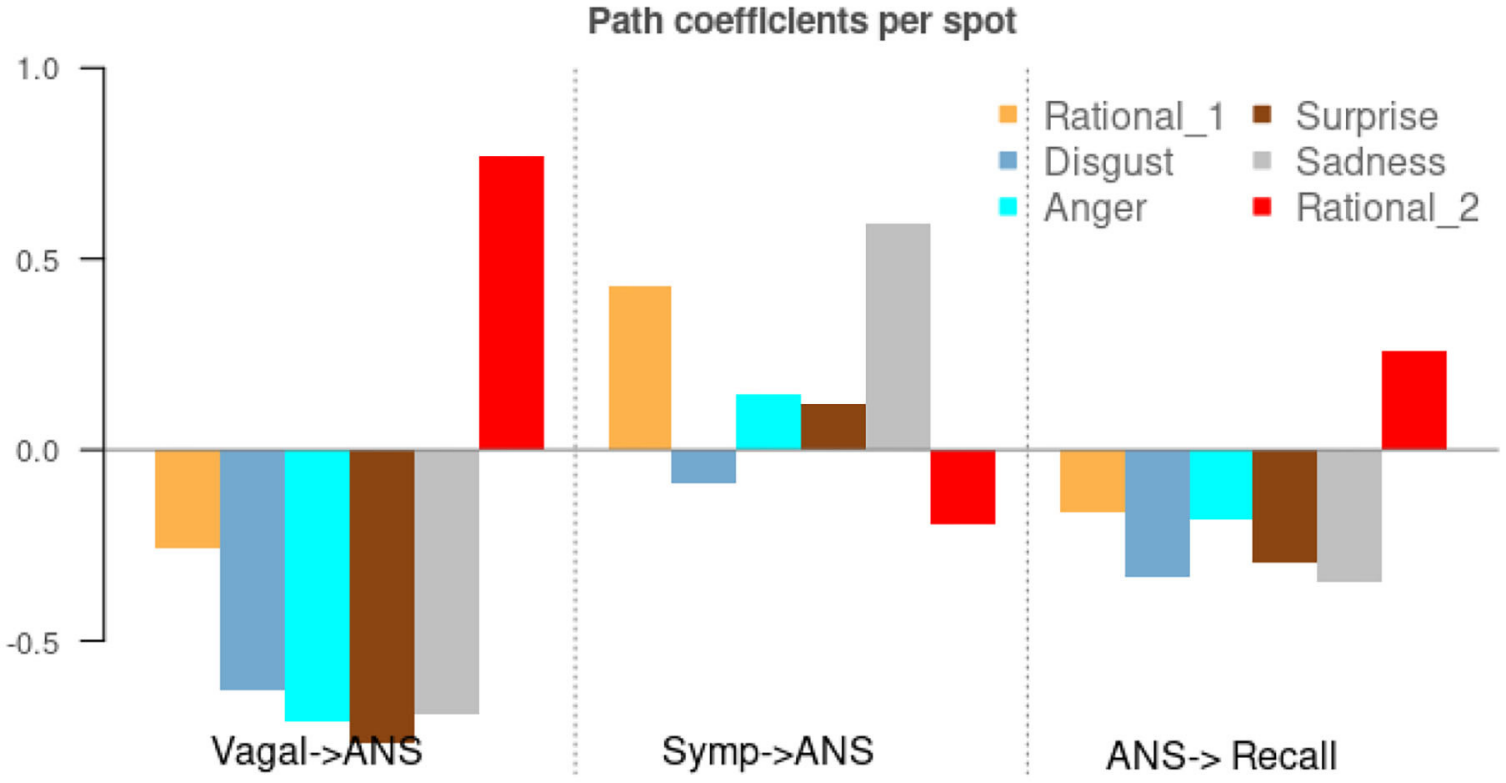
Path coefficients in the structure model comparison between emotions conveyed by the advertisements. Values in y-axes should be interpreting as standardize linear regression weights so that a negative value indicates a negative correlation and the higher the value the higher the contribution of the LV.

Figure 3 shows the path coefficients in the structure model. As shown in Figure 3, the structure model is comprised of two relational submodels: (1) the ANS as a function of the Sympathetic and Vagal activity, which is represented in Figure 3 by the two first blocks (Symp → ANS; Vagal → ANS), and (2) recall as a function of the ANS activity, third block (ANS → Recall).

The behavior is very similar to all emotions except for Rational in the last advertisement. In general, the vagal activity is negatively correlated with the ANS activity, whereas sympathetic activity positively correlated with ANS. As expected, the Vagal branch had a higher impact on ANS behavior. This behavior could be explained because it is more complicated to obtain isolated indices that account only for the Sympathetic activity. Indeed, the vagal LV was always statistically significant for every spot, whereas the sympathetic LV was only statistically significant in *Rational 1, Surprise*, and *Sadness* spots (*p*-value <0 0.05). The ANS LV was only statistically significant explaining the Recall LV in the *Sadness* spot (*p*-value < 0.05).

It is worth noting that the model's behavior for the last spot (*Rational 2*) was completely different. Indeed, this advertisement was utterly different from the others. Even though the first spot was labeled as Rational, it is about hits by cars on urban streets, and it contains real images of hits. Nonetheless, the last spot only contains speeches of doctors about the importance of rehab.

**Figure 4:**
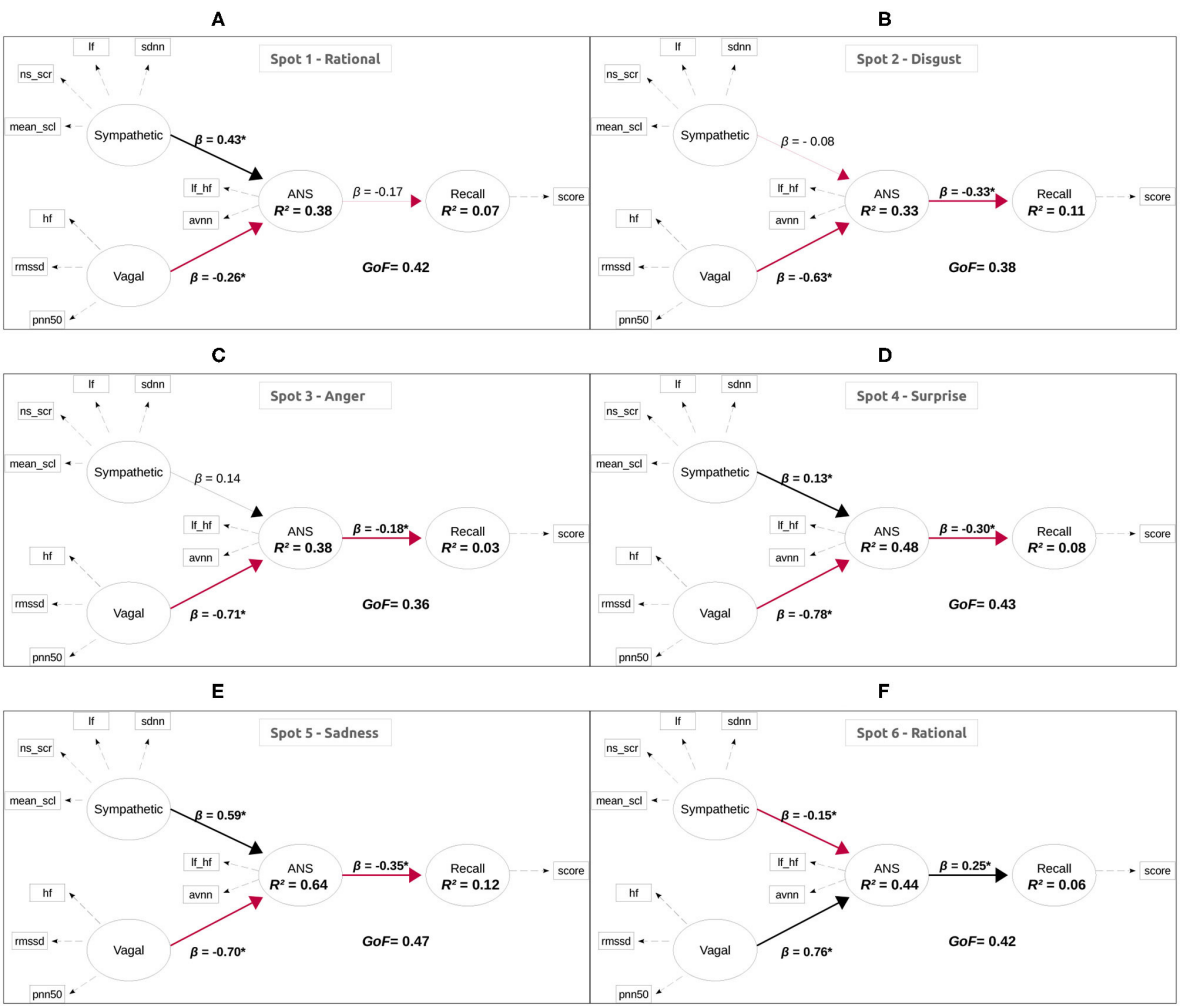
Results of fitted PLS-PM. The plots show *R*^2^, *GoF*, and β path coefficients for each spot. Significant coefficients are highlighted. Red arrows indicate a negative relationship, whereas black arrows represent a positive relationship. **(A)** Spot 1–Rational, **(B)** Spot 2–Disgust, **(C)** Spot 3–Anger, **(D)** Spot 4–Surprise, **(E)** Spot 5–Sadness, **(F)** Spot 6–Rational.

Figure 4 shows the results of the fitted PLS-PM for each spot, in particular, *R*^2^, *GoF*, and β path coefficients for the inner model. The coefficient of determination, *R*^2^ can be understood as the percentage of the variance of the ANS that is explained by the sympathetic and vagal activity, and the percentage of the variance of recall that is explained by ANS. It can be seen the different behavior of Spot 6, which induced a different relationship between LV.

The PLS-PM model captured a moderate-high variability of ANS behavior, ranging from 38% up to 64% of the explained variance of the ANS. However, the model is only able to account, at most, for 11% of the recall variance (as measured by the recall-score). The overall performance of the model is moderate, with goodness-of-fit values around 0.4 for all spots.

**Figure 5:**
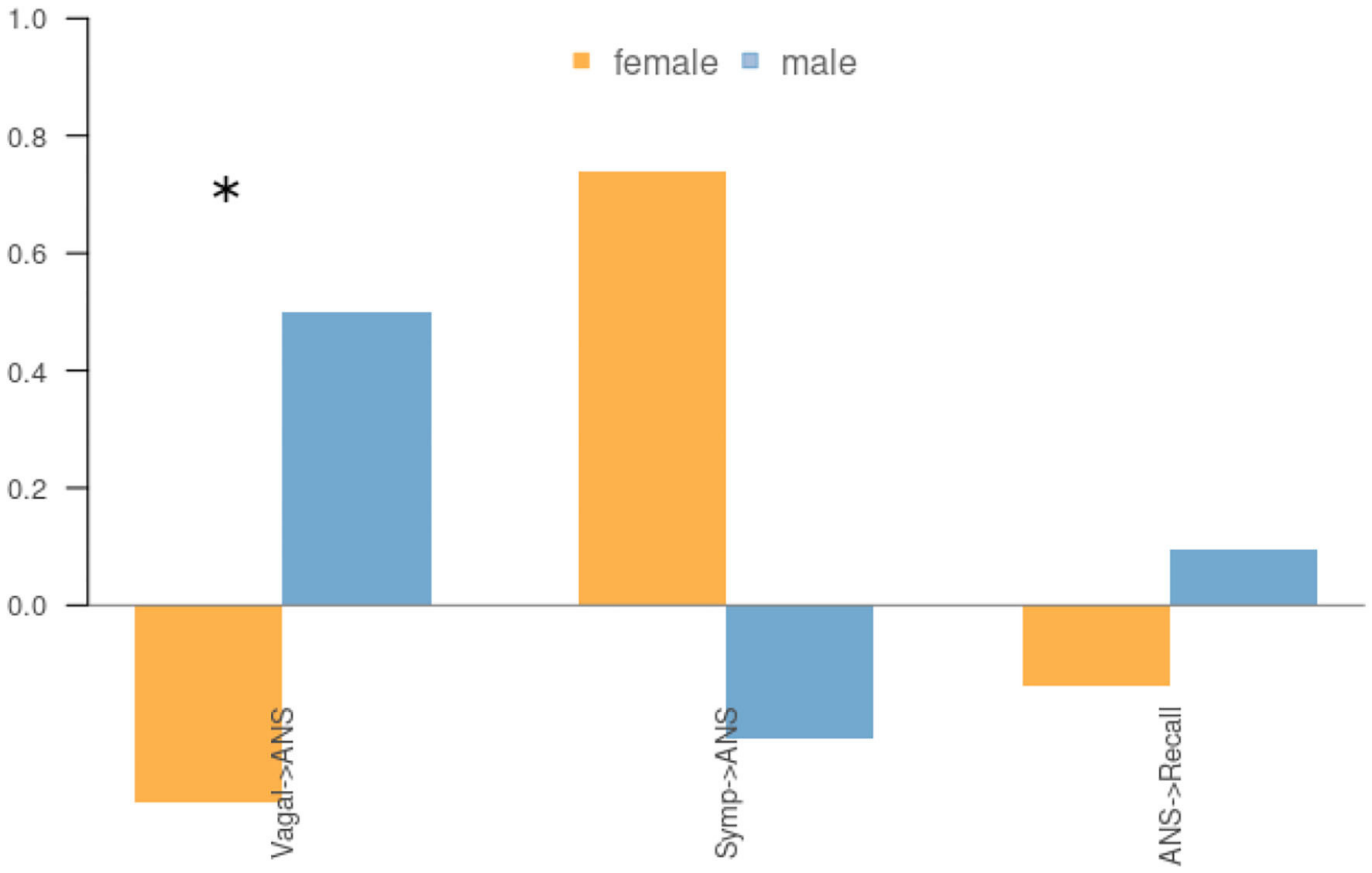
Gender differences for Spot 1 (rational) on β path coefficients. Statistically significant differences are indicated with a *.

There are no significant differences between male and females, except for Spot 1 (rational), in which the relationships between LV's is the opposite by gender, and the difference is statistically significant for the relationship between *Sympathetic* and *ANS* LVs (see Figure 5).

## 6. Discussion and Conclusions

We propose a statistical model based on PLS-PM to assess the ANS activity, as described by sympathetic and vagal branches, on subjects during advertisement observation. Subject memory formation was evaluated by filling a survey to measure recall, both spontaneous and assisted. To characterize the ANS, the ECG and EDA were measured for every subject. From these physiological signals, several autonomic indices were computed, namely, HRV time-domain and frequency-domain indices and two indices from EDA. The PLS-PM approach allows us to incorporate contextual knowledge by defining a so-called structural model composed of unobservable variables (LVs), which are constructs defined by the measured indices (MVs). In the proposed model, we set two LVs accounting for sympathetic and vagal activity. An additional LV accounts for the ANS balance, which is related to the previous LVs. Finally, the ANS is associated with a fourth LV accounting for recall.

We conducted an experiment where 66 subjects watched six advertisements while ECG and EDA were recorded. After signal quality review, a data set from 43 subjects (25 females) was available. Each advertisement conveyed a different emotion, namely: rational, disgust, anger, surprise, sadness, and rational.

The emotions caused an interaction between sympathetic and vagal activity in such a way that when the vagal tone increased, the net balance of the ANS decreased, and when the sympathetic tone increased, the net balance of the ANS increased. This interaction was quite the opposite when the advertisements were utterly rational. The recall is an abstract concept that is hard to define and more complicated to quantify. We tried to obtain a quantitative measure of the recall by giving a score based on the survey's answer. However, the model, based on the estimation of the ANS, was only able to explain 12% of the variance of this recall-score.

In this work, we proposed to model recall as a consequence of the emotional state. This modeling can only be achieved thoroughly using a combination of classical and neuromarketing techniques (Shen and Morris, 2016; Baraybar-Fernández et al., 2017). Accordingly, we used questionnaires to measure the degree of recall and neuromarketing tools to assess the emotional state through the ANS response. Our results agree with previous studies in which different induced emotions modify the ANS response (McCraty et al., 1995; Kop et al., 2011). Moreover, our results suggest that the influence of the ANS on the recall is opposite in emotion-based and fact-based advertisements (rational).

Even though HRV is a marker of the ANS control on cardiovascular activity, there are some difficulties in assessing the sympathetic activity from the HRV power spectrum accurately. Some approaches proposed to combine sympathetic information estimated from EDA and vagal information estimated from HRV, yielding a reliable sympathovagal assessment (Ghiasi et al., 2018, 2020).

Both cardiac and respiratory systems are regulated by the ANS, whose mechanisms are not fully understood and are still subject to scientific discussion (Malik and Camm, 1993; Betts et al., 2013; Reyes del Paso et al., 2013). Modulation of the heart rate by respiration, known as respiratory sinus arrhythmia, changes the RR interval duration according to the respiration cycle. Parasympathetic activity is very closely related to respiratory sinus rhythm, and in rest and healthy conditions, it generally contributes to HF power. However, under some conditions, the respiration rate may affect LF power by overestimating sympathetic activity. Furthermore, the parasympathetic activity can be underestimated when the respiratory rate is higher than the HF band (Eckberg, 2009; Karemaker, 2009). Some approaches have been published in the scientific literature trying to separate respiratory influences from the HR for a better estimate of the sympathovagal balance (Varon et al., 2018). In the present study, with healthy subjects in rest conditions, we do not consider a significant influence of the respiratory rate. However, for future works, it is worth analyzing whether the separation of respiratory influences helps knowledge acquisition related to the interactions assessed in this study.

We have proposed an easy-to-interpret model to explain the recall-score and the ANS response to emotional advertisements, which could help to design emotion-based advertisements. An improvement to this approach could be to include physiological information from the brain activity by an EEG, such as incorporating theta and alpha bands power in prefrontal cortex leads (Vecchiato et al., 2014; Cartocci et al., 2017). A limitation is that the model only accounts for linear relationships between MVs and LVs. However, it is well-known that the ANS balanced emotion mediated could have some non-linearity. A simple extension with which to account for non-linearity is the kernelized version of the PLS-PM algorithm (Tenenhaus, 2009).

## Data Availability Statement

The raw data supporting the conclusions of this article will be made available by the authors, without undue reservation.

## Ethics Statement

The studies involving human participants were reviewed and approved by Comité de Ética de la Investigación de la Universidad Rey Juan Carlos. The patients/participants provided their written informed consent to participate in this study.

## Author Contributions

ÓB-P and RG-E designed the experiment and the idea for the work, they conducted the processing of the signal, analysis of the data, and writing of the manuscript. MC-V and AV-V conducted the experiments with the subjects and gathered, curated the database, and also contributed with the writing of the manuscript. All authors contributed to the article and approved the submitted version.

## Conflict of Interest

The authors declare that the research was conducted in the absence of any commercial or financial relationships that could be construed as a potential conflict of interest.
