# Supplementary material for: Autonomic Nervous System and Recall Modeling in Audiovisual Emotion-Mediated Advertising Using Partial Least Squares-Path Modeling
Source: Front Psychol. 2020 Oct 30;11:576771. doi: 10.3389/fpsyg.2020.576771 (PMC7662410; doi:10.3389/fpsyg.2020.576771)
Supplement: Supplementary file 1 [file Data_Sheet_1.PDF]

Cuestionario estudio Neuromarketing

*Obligatorio

1. Identificador *

Recuerdo espontáneo

2. ¿Recuerdas algunos de los mensajes que transmiten estos anuncios? (se trata de la idea más destacada del anuncio, lo que quiere comunicar o transmitir el anunciante con el spot) *

3. ¿Cuáles fueron los que más te emocionaron y causaron agrado? *

¿Has sentido alguna de las siguientes emociones en alguno de ellos?

4. Tristeza *

Marca solo un óvalo.

	1	2	3	4	5	
Nada	○	○	○	○	○	Mucho

5. (Tristeza) Describe qué anuncio o qué imágenes te provocaron esta emoción *

6. Ira *

Marca solo un óvalo.

	1	2	3	4	5	
Nada	○	○	○	○	○	Mucho

7. (Ira) Describe qué anuncio o qué imágenes te provocaron esta emoción *

8. Sorpresa *

Marca solo un óvalo.

	1	2	3	4	5	
Nada	○	○	○	○	○	Mucho

9. (Sorpresa) Describe qué anuncio o qué imágenes te provocaron esta emoción *

____

10. Asco *

Marca solo un óvalo.

	1	2	3	4	5	
Nada	◯	◯	◯	◯	◯	Mucho

11. (Asco) Describe qué anuncio o qué imágenes te provocaron esta emoción *

____

12. ¿Qué secuencias/imágenes han sido más significativas para ti? *

____
____
____
____
____

Recuerdo Sugerido

13. De los siguientes temas ¿cuáles recuerdas haber visto en estos anuncios? *

Selecciona todos los que correspondan.

- [ ] Violencia de género
- [ ] Exceso de velocidad al volante
- [ ] Bullying (acoso escolar)
- [ ] Alcohol y conducción
- [ ] Machismo
- [ ] Consumo de cocaína
- [ ] Abuso infantil
- [ ] Racismo
- [ ] Ecología/desforestación
- [ ] Consumo de drogas y alcohol
- [ ] Maltrato animal
- [ ] Uso del móvil al volante
- [ ] Cáncer
- [ ] Inmigración
- [ ] Maltrato infantil
- [ ] Despistes en la carretera
- [ ] Tratamiento de adicciones
- [ ] Mobbing (acoso laboral)

Recuerdo Sugerido - De las siguientes situaciones ¿cuáles recuerdas haber visto en estos anuncios? ¿con qué temas relacionas las situaciones que recuerdas?

Un niño de cristal que se rompe al chocar con el parachoques de un coche

14. Recuerdas esta situación *

Marca solo un óvalo.

- ( ) Sí
- ( ) No

15. Temas con los que relacionas esta situación *

Selecciona todos los que correspondan.

- [ ] Violencia de género
- [ ] Exceso de velocidad al volante
- [ ] Bullying (acoso escolar)
- [ ] Alcohol y conducción
- [ ] Machismo
- [ ] Consumo de cocaína
- [ ] Abuso infantil
- [ ] Racismo
- [ ] Ecología/desforestación
- [ ] Consumo de drogas y alcohol
- [ ] Maltrato animal
- [ ] Uso del móvil al volante
- [ ] Cáncer
- [ ] Inmigración
- [ ] Maltrato infantil
- [ ] Despistes en la carretera
- [ ] Tratamiento de adicciones
- [ ] Mobbing (acoso laboral)

Otro: [ ] ____________________

Un policía hablando de los riesgos de no respetar los límites de velocidad

16. Recuerdas esta situación *

Marca solo un óvalo.

- ( ) Sí
- ( ) No

17. Temas con los que relacionas estas situación *

Selecciona todos los que correspondan.

- [ ] Violencia de género
- [ ] Exceso de velocidad al volante
- [ ] Bullying (acoso escolar)
- [ ] Alcohol y conducción
- [ ] Machismo
- [ ] Consumo de cocaína
- [ ] Abuso infantil
- [ ] Racismo
- [ ] Ecología/desforestación
- [ ] Consumo de drogas y alcohol
- [ ] Maltrato animal
- [ ] Uso del móvil al volante
- [ ] Cáncer
- [ ] Inmigración
- [ ] Maltrato infantil
- [ ] Despistes en la carretera
- [ ] Tratamiento de adicciones
- [ ] Mobbing (acoso laboral)

Otro: [ ] ____________________

Un niño que le lleva una botella de cerveza a su padre en una fiesta

18. Recuerdas esta situación *

Marca solo un óvalo.

- ( ) Sí
- ( ) No

19. Temas con los que relacionas estas situación *

Selecciona todos los que correspondan.

- [ ] Violencia de género
- [ ] Exceso de velocidad al volante
- [ ] Bullying (acoso escolar)
- [ ] Alcohol y conducción
- [ ] Machismo
- [ ] Consumo de cocaína
- [ ] Abuso infantil
- [ ] Racismo
- [ ] Ecología/desforestación
- [ ] Consumo de drogas y alcohol
- [ ] Maltrato animal
- [ ] Uso del móvil al volante
- [ ] Cáncer
- [ ] Inmigración
- [ ] Maltrato infantil
- [ ] Despistes en la carretera
- [ ] Tratamiento de adicciones
- [ ] Mobbing (acoso laboral)

Otro: [ ] ______________________

Imágenes rebobinando las escenas de un atropello

20. Recuerdas esta situación *

Marca solo un óvalo.

- ( ) Sí
- ( ) No

21. Temas con los que relacionas estas situación *

Selecciona todos los que correspondan.

- [ ] Violencia de género
- [ ] Exceso de velocidad al volante
- [ ] Bullying (acoso escolar)
- [ ] Alcohol y conducción
- [ ] Machismo
- [ ] Consumo de cocaína
- [ ] Abuso infantil
- [ ] Racismo
- [ ] Ecología/desforestación
- [ ] Consumo de drogas y alcohol
- [ ] Maltrato animal
- [ ] Uso del móvil al volante
- [ ] Cáncer
- [ ] Inmigración
- [ ] Maltrato infantil
- [ ] Despistes en la carretera
- [ ] Tratamiento de adicciones
- [ ] Mobbing (acoso laboral)

Otro: [ ] ______________________

Un hombre golpeando a una mujer mientras discuten

22. Recuerdas esta situación *

Marca solo un óvalo.

- ( ) Sí
- ( ) No

23. Temas con los que relacionas estas situación *

Selecciona todos los que correspondan.

- [ ] Violencia de género
- [ ] Exceso de velocidad al volante
- [ ] Bullying (acoso escolar)
- [ ] Alcohol y conducción
- [ ] Machismo
- [ ] Consumo de cocaína
- [ ] Abuso infantil
- [ ] Racismo
- [ ] Ecología/desforestación
- [ ] Consumo de drogas y alcohol
- [ ] Maltrato animal
- [ ] Uso del móvil al volante
- [ ] Cáncer
- [ ] Inmigración
- [ ] Maltrato infantil
- [ ] Despistes en la carretera
- [ ] Tratamiento de adicciones
- [ ] Mobbing (acoso laboral)

Otro: [ ] ____________________

Una chica pinchándose para inyectarse droga

24. Recuerdas esta situación *

Marca solo un óvalo.

- ( ) Sí
- ( ) No

25. Temas con los que relacionas estas situación *

Selecciona todos los que correspondan.

- [ ] Violencia de género
- [ ] Exceso de velocidad al volante
- [ ] Bullying (acoso escolar)
- [ ] Alcohol y conducción
- [ ] Machismo
- [ ] Consumo de cocaína
- [ ] Abuso infantil
- [ ] Racismo
- [ ] Ecología/desforestación
- [ ] Consumo de drogas y alcohol
- [ ] Maltrato animal
- [ ] Uso del móvil al volante
- [ ] Cáncer
- [ ] Inmigración
- [ ] Maltrato infantil
- [ ] Despistes en la carretera
- [ ] Tratamiento de adicciones
- [ ] Mobbing (acoso laboral)

Otro: [ ] ____________________

Un sanitario/médico hablando a cámara sobre los riesgos de las adicciones

26. Recuerdas esta situación *

Marca solo un óvalo.

- ( ) Sí
- ( ) No

27. Temas con los que relacionas estas situación *

Selecciona todos los que correspondan.

- [ ] Violencia de género
- [ ] Exceso de velocidad al volante
- [ ] Bullying (acoso escolar)
- [ ] Alcohol y conducción
- [ ] Machismo
- [ ] Consumo de cocaína
- [ ] Abuso infantil
- [ ] Racismo
- [ ] Ecología/desforestación
- [ ] Consumo de drogas y alcohol
- [ ] Maltrato animal
- [ ] Uso del móvil al volante
- [ ] Cáncer
- [ ] Inmigración
- [ ] Maltrato infantil
- [ ] Despistes en la carretera
- [ ] Tratamiento de adicciones
- [ ] Mobbing (acoso laboral)

Otro: [ ] ______________________

Unos niños peleando en el patio de un colegio

28. Recuerdas esta situación *

Marca solo un óvalo.

( ) Sí

( ) No

29. Temas con los que relacionas estas situación *

Selecciona todos los que correspondan.

- [ ] Violencia de género
- [ ] Exceso de velocidad al volante
- [ ] Bullying (acoso escolar)
- [ ] Alcohol y conducción
- [ ] Machismo
- [ ] Consumo de cocaína
- [ ] Abuso infantil
- [ ] Racismo
- [ ] Ecología/desforestación
- [ ] Consumo de drogas y alcohol
- [ ] Maltrato animal
- [ ] Uso del móvil al volante
- [ ] Cáncer
- [ ] Inmigración
- [ ] Maltrato infantil
- [ ] Despistes en la carretera
- [ ] Tratamiento de adicciones
- [ ] Mobbing (acoso laboral)

Otro: [ ] ____________________

Disparo en la cabeza a una pareja en un coche

30. Recuerdas esta situación *

Marca solo un óvalo.

- ( ) Sí
- ( ) No

31. Temas con los que relacionas estas situación *

Selecciona todos los que correspondan.

- [ ] Violencia de género
- [ ] Exceso de velocidad al volante
- [ ] Bullying (acoso escolar)
- [ ] Alcohol y conducción
- [ ] Machismo
- [ ] Consumo de cocaína
- [ ] Abuso infantil
- [ ] Racismo
- [ ] Ecología/desforestación
- [ ] Consumo de drogas y alcohol
- [ ] Maltrato animal
- [ ] Uso del móvil al volante
- [ ] Cáncer
- [ ] Inmigración
- [ ] Maltrato infantil
- [ ] Despistes en la carretera
- [ ] Tratamiento de adicciones
- [ ] Mobbing (acoso laboral)

Otro: [ ] ______________________

Un grupo de niños insultando a un compañero en el colegio

32. Recuerdas esta situación *

Marca solo un óvalo.

- ( ) Sí
- ( ) No

33. Temas con los que relacionas estas situación *

Selecciona todos los que correspondan.

- [ ] Violencia de género
- [ ] Exceso de velocidad al volante
- [ ] Bullying (acoso escolar)
- [ ] Alcohol y conducción
- [ ] Machismo
- [ ] Consumo de cocaína
- [ ] Abuso infantil
- [ ] Racismo
- [ ] Ecología/desforestación
- [ ] Consumo de drogas y alcohol
- [ ] Maltrato animal
- [ ] Uso del móvil al volante
- [ ] Cáncer
- [ ] Inmigración
- [ ] Maltrato infantil
- [ ] Despistes en la carretera
- [ ] Tratamiento de adicciones
- [ ] Mobbing (acoso laboral)

Otro: [ ] ______________________

Choque de dos coches mientras vemos al niño que va en el asiento trasero de uno de ellos

34. Recuerdas esta situación *

Marca solo un óvalo.

- ( ) Sí
- ( ) No

35. Temas con los que relacionas estas situación *

Selecciona todos los que correspondan.

- [ ] Violencia de género
- [ ] Exceso de velocidad al volante
- [ ] Bullying (acoso escolar)
- [ ] Alcohol y conducción
- [ ] Machismo
- [ ] Consumo de cocaína
- [ ] Abuso infantil
- [ ] Racismo
- [ ] Ecología/desforestación
- [ ] Consumo de drogas y alcohol
- [ ] Maltrato animal
- [ ] Uso del móvil al volante
- [ ] Cáncer
- [ ] Inmigración
- [ ] Maltrato infantil
- [ ] Despistes en la carretera
- [ ] Tratamiento de adicciones
- [ ] Mobbing (acoso laboral)

Otro: [ ] ____________________

Un conductor enviando un mensaje con el móvil mientras conduce

36. Recuerdas esta situación *

Marca solo un óvalo.

- ( ) Sí
- ( ) No

37. Temas con los que relacionas estas situación *

Selecciona todos los que correspondan.

- [ ] Violencia de género
- [ ] Exceso de velocidad al volante
- [ ] Bullying (acoso escolar)
- [ ] Alcohol y conducción
- [ ] Machismo
- [ ] Consumo de cocaína
- [ ] Abuso infantil
- [ ] Racismo
- [ ] Ecología/desforestación
- [ ] Consumo de drogas y alcohol
- [ ] Maltrato animal
- [ ] Uso del móvil al volante
- [ ] Cáncer
- [ ] Inmigración
- [ ] Maltrato infantil
- [ ] Despistes en la carretera
- [ ] Tratamiento de adicciones
- [ ] Mobbing (acoso laboral)

Otro: [ ] ____________________

Una chica joven robando en una tienda

38. Recuerdas esta situación *

Marca solo un óvalo.

- ( ) Sí
- ( ) No

39. Temas con los que relacionas estas situación *

Selecciona todos los que correspondan.

- [ ] Violencia de género
- [ ] Exceso de velocidad al volante
- [ ] Bullying (acoso escolar)
- [ ] Alcohol y conducción
- [ ] Machismo
- [ ] Consumo de cocaína
- [ ] Abuso infantil
- [ ] Racismo
- [ ] Ecología/desforestación
- [ ] Consumo de drogas y alcohol
- [ ] Maltrato animal
- [ ] Uso del móvil al volante
- [ ] Cáncer
- [ ] Inmigración
- [ ] Maltrato infantil
- [ ] Despistes en la carretera
- [ ] Tratamiento de adicciones
- [ ] Mobbing (acoso laboral)

Otro: [ ] ____________________

Un joven esnifando parte de su cerebro en un lavabo

40. Recuerdas esta situación *

Marca solo un óvalo.

- ( ) Sí
- ( ) No

41. Temas con los que relacionas estas situación *

Selecciona todos los que correspondan.

- [ ] Violencia de género
- [ ] Exceso de velocidad al volante
- [ ] Bullying (acoso escolar)
- [ ] Alcohol y conducción
- [ ] Machismo
- [ ] Consumo de cocaína
- [ ] Abuso infantil
- [ ] Racismo
- [ ] Ecología/desforestación
- [ ] Consumo de drogas y alcohol
- [ ] Maltrato animal
- [ ] Uso del móvil al volante
- [ ] Cáncer
- [ ] Inmigración
- [ ] Maltrato infantil
- [ ] Despistes en la carretera
- [ ] Tratamiento de adicciones
- [ ] Mobbing (acoso laboral)

Otro: [ ] ______________________

Un padre pegando a un niño en la calle

42. Recuerdas esta situación *

Marca solo un óvalo.

- ( ) Sí
- ( ) No

43. Temas con los que relacionas estas situación *

Selecciona todos los que correspondan.

- [ ] Violencia de género
- [ ] Exceso de velocidad al volante
- [ ] Bullying (acoso escolar)
- [ ] Alcohol y conducción
- [ ] Machismo
- [ ] Consumo de cocaína
- [ ] Abuso infantil
- [ ] Racismo
- [ ] Ecología/desforestación
- [ ] Consumo de drogas y alcohol
- [ ] Maltrato animal
- [ ] Uso del móvil al volante
- [ ] Cáncer
- [ ] Inmigración
- [ ] Maltrato infantil
- [ ] Despistes en la carretera
- [ ] Tratamiento de adicciones
- [ ] Mobbing (acoso laboral)

Otro: [ ] ______________________

Este contenido no ha sido creado ni aprobado por Google.

Google Formularios
