# Supplementary material for: Autonomic Nervous System and Recall Modeling in Audiovisual Emotion-Mediated Advertising Using Partial Least Squares-Path Modeling
Source: Front Psychol. 2020 Oct 30;11:576771. doi: 10.3389/fpsyg.2020.576771 (PMC7662410; doi:10.3389/fpsyg.2020.576771)
Supplement: Supplementary file 2 [file Data_Sheet_2.PDF]

Link to the spots videos

https://drive.google.com/file/d/1XxhY3d-59ocUy7j-mICSk2Zxfxyr6AxY/view?usp=sharing
